# Negative Allosteric Modulators of mGlu_7_ Receptor as Putative Antipsychotic Drugs

**DOI:** 10.3389/fnmol.2018.00316

**Published:** 2018-09-20

**Authors:** Paulina Cieślik, Monika Woźniak, Katarzyna Kaczorowska, Piotr Brański, Grzegorz Burnat, Agnieszka Chocyk, Bartosz Bobula, Piotr Gruca, Ewa Litwa, Agnieszka Pałucha-Poniewiera, Agnieszka Wąsik, Andrzej Pilc, Joanna Wierońska

**Affiliations:** Institute of Pharmacology, Polish Academy of Sciences, Krakow, Poland

**Keywords:** schizophrenia, metabotropic glutamate receptor 7, antipsychotic, negative allosteric modulators, MMPIP, ADX71743

## Abstract

The data concerning antipsychotic-like activity of negative allosteric modulators (NAMs)/antagonists of mGlu_7_ receptors are limited. The only available ligands for this receptor are MMPIP and ADX71743. In the present studies, we used stable cell line expressing mGlu_7_ receptor and it was shown that both compounds dose-dependently potentiated forskolin elevated cAMP concentration in the T-REx 293 cells, showing their inverse agonist properties. Subsequently, pharmacokinetic studies were performed. Both compounds were given intraperitoneally (i.p.) at the dose of 10 mg/kg and reached Cmax 0.25–0.5 h after administration, and then they declined rapidly, ADX71743 being almost undetectable 2 h after administration, while the concentration of MMPIP was still observed, suggesting that the concentration of MMPIP was more stable. Finally, we investigated the role of both mGlu_7_ receptor NAMs in animal models of schizophrenia. Behavioral tests commonly used in antipsychotic drug discovery were conducted. Both tested compounds dose-dependently inhibited MK-801-induced hyperactivity (MMPIP at 15 mg/kg; ADX at 5 and 15 mg/kg) and DOI-induced head twitches (MMPIP at 5, 10, 15 mg/kg; ADX at 2.5, 5, 10 mg/kg). Moreover, the same effects were noticed in novel object recognition test, where MMPIP (5, 10, 15 mg/kg) and ADX71743 (1, 5, 15 mg/kg) reversed MK-801-induced disturbances. In the social interaction test, antipsychotic activity was observed only for ADX71743 (5, 15 mg/kg). ADX71743 at the dose 2.5 mg/kg reversed MK-801-induced disruption in prepulse inhibition while MMPIP at 10 mg/kg reversed MK-801-induced disruption in spatial delayed alternation. The present studies showed that mGlu_7_ receptor may be considered as a putative target for antipsychotic drugs, though more studies are needed due to limited number of available ligands.

## Introduction

Metabotropic glutamate receptors (mGluR) are being extensively studied as new pharmacological targets for central nervous system (CNS) disorders such as depression (Mitsukawa et al., 2006), anxiety (Swanson et al., 2005), schizophrenia (Conn et al., 2009; Nickols and Conn, 2014), neurodegenerative disorders (Gu et al., 2014; Litim et al., 2017), and pain (Acher and Goudet, 2015; Chiechio, 2016). Among these receptors, mGluR_7_ is one of the most conserved mGluR which is abundantly expressed in the cerebral cortex (Ohishi et al., 1995; Kinoshita et al., 1998; Dalezios et al., 2002), hippocampus (Ohishi et al., 1995; Kinoshita et al., 1998; Sansig et al., 2001), amygdala (Ohishi et al., 1995; Kinoshita et al., 1998), and basal ganglia (Ohishi et al., 1995; Kinoshita et al., 1998; Kosinski et al., 1999). mGluR_7_ is localized both presynaptically, where it negatively regulates glutamate and GABA release, and postsynaptically (Schoepp, 2001), where it mediates slow postsynaptic potentials. The affinity of mGlu_7_ for glutamate is also relatively low (*K*_i_ = 869 μM) (Wright et al., 2000) and thus the receptor might play a modulatory role in the CNS, protecting from glutamate overstimulation (Niswender and Conn, 2010). Recent evidence suggests mGlu_7_ receptor involvement in the pathology of schizophrenia, as several polymorphisms of gene encoding this receptor have been found in different populations, for example, significant transmission distortion of rs17031835 in intron 1 of GRM7 in Indonesian sib-pair families (Ganda et al., 2009), 14 single nucleotide polymorphisms (SNPs) in GRM7 of Han Chinese population (Li et al., 2016) or synonymus polymorphism (371T/C, rs3749380) in exon 1 of GRM7 in Japanese patients with schizophrenia (Ohtsuki et al., 2008). Due to the lack of highly specific, bioavailable compounds mGluR_7_ activity is yet poorly understood, especially in the context of schizophrenia.

Mitsukawa et al. described the first selective positive allosteric modulator of mGlu_7_ receptors – AMN082 (Mitsukawa et al., 2005). It was shown that AMN082 possesses antidepressant-like profile in FST and TST, and anxiolytic properties in four plate test and stress-induced hyperthermia (Palucha et al., 2007; Stachowicz et al., 2008). However, it did not exhibit any antipsychotic-like profile and rather enhanced MK-801- or DOI-induced effects, which may suggest a potential beneficial role of antagonists or negative allosteric modulators (NAMs) in animal models of schizophrenia (Wierońska et al., 2012). Additionally, some studies reported off target activity of AMN082 (Sukoff Rizzo et al., 2011).

Up to date, there have been only two mGlu_7_ receptor NAMs synthetized – MMPIP and ADX71743 (Suzuki et al., 2007; Kalinichev et al., 2013). MMPIP was shown to impair cognition and decrease social interaction in WT mice or rats, bared no effect on spontaneous activity and motor performance (Hikichi et al., 2010) but induced analgesic effects (Palazzo et al., 2016, 2015). Additionally, no antidepressive and anxiolytic effects were described. Moreover, MMPIP did not reverse the pharmacologically induced disruption of prepulse inhibition (PPI, Hikichi et al., 2010). However, the effects of MMPIP might be difficult to explain as it also acts as an inverse agonist (Suzuki et al., 2007).

Another NAM of mGlu_7_ receptor – ADX71743 – was found to exert an anxiolytic but not antidepressant effect (Kalinichev et al., 2013). When administered to animals, it did not impair their locomotor activity and motor performance. The antipsychotic activity of ADX71743 is not well described and understood as it caused a moderate decrease in amphetamine-induced hyperactivity, but had no effect on DOI-induced head twitches and the conditioned avoidance response (Kalinichev et al., 2013).

Here, we extensively describe the action of MMPIP and AXD71743 both *in vitro* and *in vivo* in the context of schizophrenia. Antipsychotic activity of both compounds was evaluated in number of behavioral tests, such as: MK-801-induced hyperactivity, DOI-induced head twitches, modified forced swim test, social interaction test, PPI, and novel object recognition (NOR) test. Their potential effect on motor performance was assessed in rotarod test. Due to better pharmacokinetic properties, the activity of MMPIP was also tested in spatial delayed alternation test. In order to confirm the profile of interaction of MMPIP and ADX71743 with mGlu_7_ receptor, the intracellular levels of cAMP were measured. Additionally, pharmacokinetic and electrophysiological studies were performed.

## Materials and Methods

### Animals and Housing

Male Albino Swiss mice (20–25 g) were used in most behavioral tests. Male Wistar rats (200–250 g) were used in spatial delayed alternation test and PPI of the acoustic startle response test. Male C57BL/6J WT and mGlu_7_ KO mice were used in electrophysiological studies. The animals were kept in a room with 12:12 light–dark cycle at a temperature of 21–22°C. Food and water were provided *ad libitum*. The animals were used only once, none of the animals has been run multiple experiments. All procedures were conducted according to the guidelines of the National Institutes of Health Animal Care and Use Committee and were approved by the II Local Ethics Committee by the Institute of Pharmacology, Polish Academy of Sciences in Krakow.

### Drugs

MMPIP [6-(4-Methoxyphenyl)-5-methyl-3-(4-pyridinyl)-isoxazolo(4,5-c)pyridin-4(5H)-one], ADX71743 [6-(2,4-Dimethylphenyl)-2-ethyl-6,7-dihydro-4(5*H*)-benzoxazolone], MK-801 [(5*S*,10*R*)-(+)-5-Methyl-10,11-dihydro-5*H*-dibenzo[*a,d*]cyclo-hepten-5,10-imine maleate] and DOI (4-Iodo-2,5-dimethoxy-α-methylbenzeneethanamine hydrochloride) were purchased from Tocris Bioscience, Bristol, United Kingdom. For behavioral and pharmacokinetic studies, MK-801 and DOI were dissolved in 0.9% NaCl, MMPIP in 0.5% methylcellulose (Sigma–Aldrich, St Louis, MO, United States) and ADX71743 in small amount of DMSO (Sigma–Aldrich) and then titrated in 20% captisol (Cydex Pharmaceuticals, Lawrence, KS, United States). Final concentration of DMSO in the whole solution was 2%. Control groups received appropriate vehicles. All drugs were administered in a volume of 10 ml/kg when given to mice, and 1 ml/kg when given to rats. The doses of the compounds we used were partially chosen on the basis of the other studies (Hikichi et al., 2010), but mostly were established experimentally. Mostly, the compounds were administered up to the dose of 15 mg/kg; however, in some case when the activity was evident at the lower doses, the dose of 15 mg/kg was not investigated.

### cAMP

A homogeneous time-resolved fluorescence (HTRF) cAMP dynamic 2 (Cisbio, Codolet, France) assay was performed as previously described (Chruścicka et al., 2015) with recombinant cell lines. Briefly, HEK 293 T-REx cells stably expressing mGlu_7_ receptor, were collected and suspended in Hanks-HEPES buffer. The cell suspension was added to compounds solution with 5 μM of forskolin (final concentration). After 5 min incubation in 37°C, 5 μl of cAMP-d2 conjugate in lysis buffer was added and mixed with the 10 μl cell suspension by means of an automated pipetting system (Tecan Evo 200, Tecan, Mannedorf, Switzerland). Next, 5 μl anti-cAMP cryptate conjugate was added and the fluorescence at 620 and 665 nm was read after 1 h (Tecan Infinite M1000). The results are shown as the 665 nm/620 nm ratio multiplied by 10^4^. The detected signal was inversely proportional to the concentration of cAMP in the sample. Antagonist activity of ADX71743 or MMPIP are shown as a percentage of the inhibition of L-Glu activity at its EC_80_ concentration. Dose response data from ADX71743 or MMPIP were analyzed with Prism Version 7.03 (GraphPad Software Inc.). Each experiment was performed three times (*n* = 3), and each data point was in triplicate.

### Pharmacokinetic Studies

The method described below was successfully applied to a pharmacokinetic study of ADX71743 and MMPIP in mouse (Albino Swiss) after i.p. injection. Compound ADX71743 and MMPIP were administered to mice at 10 mg/kg i.p. At 0.25, 0.50, 1.0, 2.0, 4.0, 6.0 h, the mice were anesthetized, and the blood was collected from the portal vein to the tubes containing 5% EDTA. The mice were then perfused with 0.1M PBS to remove remaining blood from the body, and the brains were taken out for the analysis. Blood was centrifuged at 2000 rpm for 10 min at 4°C, and the plasma was collected and frozen at -80°C for further analysis.

Plasma and tissue samples from all drug-treated animals were thawed at room temperature prior to use. Standard protocol of sample preparation: 200 μl acetonitrile was added to the eppendorfs with 50 μl of studied plasma samples or tissue homogenate. Samples were mixed for 5 min on a mixer at 25°C and 1400 rpm. Tubes were then centrifuged at 2000 × *g* for 15 min at 4°C. About 180 μl of each supernatant was transferred into a plate well. Finally, each sample was injected into the column.

In calibration curve – serial dilution method, plasma was spiked with standard at different concentration levels. Acetonitrile was added. Mixed, centrifuged supernatant was taken.

### LC-MS Analysis

#### Chromatographic Conditions

Plasma and tissue samples from all drug-treated animals at selected time points are analyzed using previously developed a non-validated liquid chromatography-tandem mass spectrometry (LC-MS)/MS method. A sensitive and highly selective LC-MS method was used to determine drug concentration in mouse plasma samples or tissue homogenate.

LC/MS analysis was carried on a Bruker amaZon SL mass spectrometer using positive/ negative ion ESI mode. Chromatographic separation was achieved on a Ascentis Express C_18_ column, (5 cm × 2.1 mm, 2.7 μM, Supelco Technologies) at room temperature with a thermostatted column oven. A gradient elution of eluents A [acetonitrile (LiChrosolv, Reag. Ph Eur) +0.1% formic acid (Sigma–Aldrich, 98–100%)] and B (water +0.1% formic acid) was used for separation. The flow rate was set at 1 ml/min. The injection volume was 20 μl, and the time of injection was 4 min.

#### Mass Spectrometric Conditions

An Ion trap mass spectrometer (Bruker amaZon SL) was equipped with an electrospray source, operating in the positive/negative ion mode. Data were collected and processes using Bruker Quant Analysis software. Quantification of analytes was performed in SIM mode.

### Electrophysiology

Mice (wild and KO, approx. 25 g) were housed under a controlled light/dark cycle (light on: 0700–1900) and had free access to standard food and tap water. Mice were anesthetized with isoflurane (Aerrane, Baxter) decapitated, their brains were dissected and immersed in an ice-cold artificial cerebrospinal fluid (ACSF) of the following composition (in mM): NaCl (130), KCl (5), CaCl_2_ (2.5), MgSO_4_ (1.3), KH_2_PO_4_ (1.25), NaHCO_3_ (26), and glucose (10), bubbled with a mixture of 95% O_2_/5% CO_2_ to pH 7.4. Frontal cortical slices (bregma 1.9–1.4, 380 μm) were cut in a coronal plane using a vibrating microtome and they were stored at 32°C. A single slice was next transferred to the recording chamber (32°C ± 0.5°C) and superfused at 2.5 ml/min with a ACSF.

**FIGURE 1:**
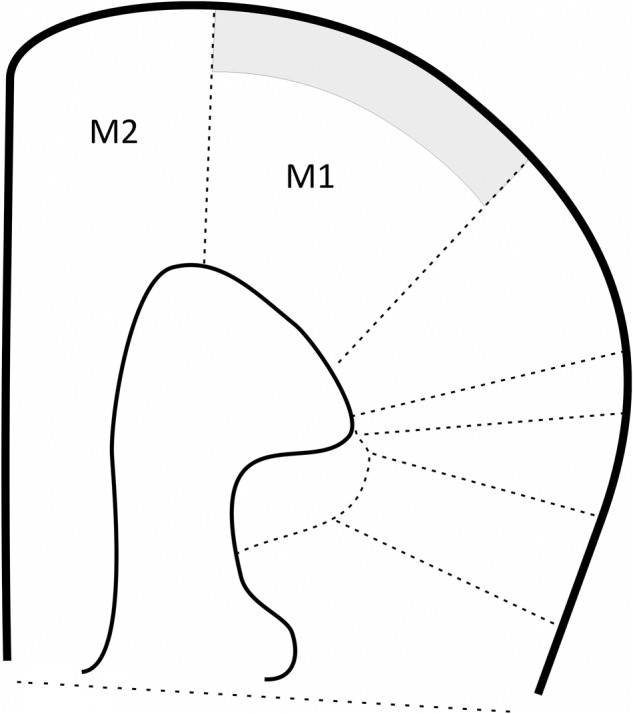
Schematic illustration showing mice cortex coronal slice (Paxinos and Franklin, 2012). Area colored in gray represents recording zone.

A bipolar stimulating electrode (FHC) was placed approx. 2 mm lateral to the midline and approx. 1.0 mm below the pial surface (in layer V) (**Figure 1**). Stimuli (duration: 0.2 ms) were applied at 0.033 Hz using a constant-current stimulus isolation unit (WPI). Field potentials (FPs) were recorded using glass micropipettes filled with ACSF (1–3 MΩ), which were placed approx. 0.2 mm below the cortical surface (in layer II/III). FPs were amplified (Axoprobe 1A, Axon Instruments), A/D converted at 10 kHz and stored using Micro1401 interface and Signal 4 software (CED).

The stimulus–response curves obtained for each slice were fit with the Boltzmann equation: Vi = Vmax/(1 + exp[(u - Uh)/-S]), where Vmax is the maximum FP amplitude; u is the stimulation intensity; Uh is the stimulation intensity evoking FP of half-maximum amplitude; S is the factor proportional to the slope of the curve; “exp” is exponentiation – mathematical operation. The results are expressed as the means ± SEM. Statistical analyses were carried out using *t*-test (Tokarski et al., 2011).

For each slice, at the beginning of the experiment an input-output curve was generated in ACSF. A stimulus-response (input-output) curve was made for each slice. To obtain the curve, stimulation intensity was gradually increased stepwise (16 steps; 0–100 μA). One response was recorded at each stimulation intensity. Next, standard ACSF was replaced by a solution containing MMPIP or ADX71743, for 20 min, and input-output curves were generated again. Statistical analyzes were carried out using paired *t*-test and ANOVA.

### MK-801-Induced Hyperactivity

The locomotor activity was recorded individually for each animal in OPTO-M3 locomotor activity cages (Columbus Instrument) linked online to a compatible PC activity, as described previously by Woźniak et al., 2016b. Each cage (13 cm × 23 cm × 15 cm) was surrounded with an array of photocell beams. Interruptions of these photobeams resulted in horizontal activity defined as ambulation counts. The mice were placed in the locomotor activity cages for acclimatization for 30 min Then, MMPIP (10, 15 mg/kg) or ADX71743 (5, 10 mg/kg) were administered i.p. Both drugs were given 30 min prior to MK-801 injection (0.35 mg/kg, i.p.). The locomotor activity was measured for 60 min immediately after MK-801 administration.

### DOI-Induced Head Twitches

The experiment was performed according to previously described procedure (Wierońska et al., 2012, 2013). Immediately after a 30 min acclimatization period, DOI (2.5 mg/kg, i.p.) was administered in order to induce head twitches. The number of head twitches was then counted for 20 min. MMPIP (5, 10, and 15 mg/kg) or ADX71743 (2.5, 5, and 10 mg/kg) were administered i.p. 30 min before DOI. Subsequently the compounds were administered chronically (for 10 days) each at the two active doses (MMPIP 5 and 10 mg/kg and ADX71743 2.5 and 5 mg/kg). The test was performed on 11th day, 30 min after the last administration.

### Modified Forced Swim Test

The modified forced swim test was performed according to the method introduced by Noda (Noda et al., 1995, 1997; Wierońska et al., 2015a; Woźniak et al., 2016a). The swim tests were performed in a glass cylinder (height, 20 cm; internal diameter, 15 cm) containing 11 cm of water maintained at 23–24°C. After the acclimation period, the animals underwent the first swim test, where the immobility time was measured during a 3 min period (T_1_). On the next day, chronic (13 days) MK-801 administration (0.4 mg/kg, i.p.) was started. After a 1-day break, on the 15th day of experiment, the second swim session was performed and the immobility time during 3-min test was measured again (T_2_). The T_2_ - T_1_ difference was reported as the result of the experiment. MMPIP (1, 5, and 15 mg/kg, i.p.) or ADX71743 (5, 10, and 15 mg/kg, i.p.) were administered acutely 30 min before the T_2_ session.

### Social Interaction Test

The method was adapted from de Moura Linck et al., 2008 and Woźniak et al., 2016b. After the 2-day habituation trial (10 min/day) a pair of mice was placed in the open field for 5 min. The social interactions between two mice were determined based on the total time spent participating in social behavior such as genital investigation, sniffing, chasing, and fighting each other. The total number of social episodes was also measured. The test was video-recorded and viewed by a trained observer. MMPIP (5, 10, and 20 mg/kg, i.p) or ADX71743 (1, 5, and 15 mg/kg, i.p.) were administered 30 min before MK-801 (0.3 mg/kg, i.p.), which was administered 30 min before the test.

### Novel Object Recognition Test

The experiment was performed according to Nilsson et al., 2007 with minor modifications (Woźniak et al., 2016b). Following a 2-day habituation period (10 min/day), a training trial was performed, where mice were allowed to explore two identical objects for 5 min. About 1 h later, a test trial was conducted, where one of the familiar object was replaced by a novel object. The animals were then allowed to explore the objects for 5 min. MMPIP (5, 10, and 15 mg/kg, i.p.) and ADX71743 (1, 5, and 10 mg/kg; i.p.) were administered 30 min before MK-801 (0.3 mg/kg, i.p.), which was administered 30 min before the training trial. Time spend exploring (i.e., sniffing or touching) the familiar (T_familiar_) or novel object (T_novel_) was measured by a trained observer and then the recognition index was calculated for each mouse [(T_novel_ - T_familiar_)/(T_familiar_ + T_novel_)] × 100.

### Rotarod Test

The animals were trained for 3 consecutive days at the speed of 18 rpm, one session per day for 3 min. If a mice fell during the habituation period, it was placed back on the apparatus. On the following day, the test trial was performed. After the mice were placed on the apparatus (Mouse Rota-Rod NG, UGO BASILE S.R.L.) moving at the speed of 12 rpm, the accelerating mode was started (maximum speed – 24 rpm). The latency to fall was measured during 3-min test session. Mice were injected with MMPIP (5, 15, and 30 mg/kg, i.p.) or ADX71743 (5, 15, and 30 mg/kg, i.p.) 30 min before the test trial.

### Spatial Delayed Alternation Test

The spatial delayed alternation test was performed using a wooden T-maze, according to Sławińska et al., 2013 and Wierońska et al., 2015b.

During the adaptation phase, lasting 3 days, the animals were allowed to freely explore the maze for 10 min. For the next 2 days, rats were confined to either of the two end-arms and allowed to drink a 10 % sucrose solution there for 10 min twice daily. On the following day, a 2-week training phase was started. The animals performed one training session per day, which consisted of one forced trial (i.e., one of the end-arms was closed) followed by ten free choice trials. During the free choice trial the animal was placed in the starting arm and after the guillotine door was raised, it was allowed to choose to enter one of the end-arms. After the response, the rat was placed back to the starting arm, where it stayed for 10 s. If the chosen end-arm was the opposite to the previously visited one, a correct response was scored, and the animal was closed in the compartment where it was allowed to drink the sucrose solution for 5 s. After an incorrect response, the animal was gently returned to the starting arm. The training phase was carried out until the animals scored 7 correct responses in a training session in 2 consecutive days.

On the day of the test, the animals were injected with MMPIP and/or MK-801, and the aforementioned 10-trial session was repeated. MMPIP was administered at a dose of 5 or 10 mg/kg 30 min prior to MK-801 (0.1 mg/kg) administration. The test was started 30 min after the MK-801 injection.

### Prepulse Inhibition

The procedure was performed according to Czyrak et al., 2003. On the day before the experiment, the animals were subjected to a single startle session consisting of two trials, each presented 20 times during the session. During the first trial, a 120 dB, 40 ms pulse was presented, and on the second trial this pulse was preceded by a 75 dB, 20 ms prepulse. On the day of the experiment, the animals were habituated to the background white noise (65 dB) for 5 min (which continued throughout the test), after that the startle session was carried out as described above. Startle response amplitude was defined as the difference between the maximum force detected during a recording window and the force measured immediately before the stimulus onset (the threshold was set at 10 g). For each animal, the amplitudes were averaged separately for each type of trial. The PPI was calculated as the difference between the amplitudes of the pulse (P) and the prepulse + pulse (PP+P), divided by the amplitude of the pulse alone [([P - (PP + P)]/P)×100].

MMPIP (5, 10, and 15 mg/kg) and ADX71743 (2.5, 5, and 10 mg/kg) were administered 30 min prior to MK-801 (0.3 mg/kg), which was administered 30 min before the habituation phase.

### Statistical Analysis

Statistical analysis was performed using Statistica 12 package (StatSoft Inc., Tulsa, OK, United States). A one-way ANOVA followed by Newman-Keuls *post hoc* test was used to analyze the behavioral experiments and Student’s *t*-test for paired samples was used to assess the differences in the amplitude of FPs. Data are presented as mean ± SEM.

## Results

### cAMP

**FIGURE 2:**
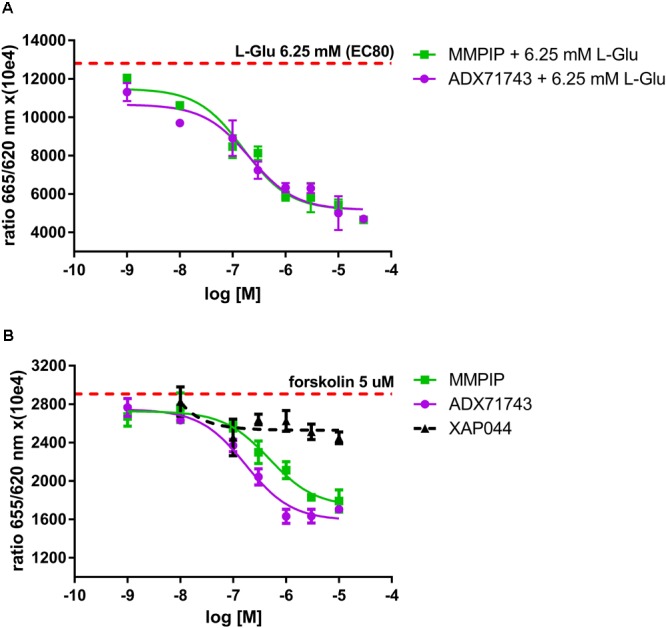
MMPIP and ADX71743 antagonized L-Glu inhibition of cAMP accumulation in presence of 5 μM of forskolin thus confirming their NAM profile **(A)** MMPIP and ADX71743 enhance the action of forskolin in dose-dependent manner increasing cAMP concentration. This effect was not observed for XAP044. The dashed line represents the cAMP level corresponding to 5 μM of forskolin **(B)**. Representative results, data points presented as mean ± SEM.

In order to confirm the NAM profile of ADX71743 and MMPIP, the substances were incubated with 6.26 mM of L-Glu (EC_80_). Both ADX71743 and MMPIP dose-dependently antagonized L-Glu inhibition of cAMP accumulation in the presence of forskolin, with the IC_50_ values of 0.44 μM (± 0.13) (n = 3) and 0.38 μM (± 0.15) (n = 3) respectively (**Figure 2A**).

In the second set of experiments the cells were incubated with forskolin with increasing concentration of ADX71743 or MMPIP without agonist in order to analyze their inverse agonist properties. Both compounds dose-dependently potentiated forskolin action, elevating cAMP concentration in the T-REx 293 cells (**Figure 2B**). IC_50_ of both substances was very similar – ADX71743 0.22 μM (± 0.07) (n = 3) and MMPIP 0.34 μM (± 0.14) (n = 3). This effect was not observed for an antagonist of mGluR_7_ – XAP044.

### Pharmacokinetics

**FIGURE 3:**
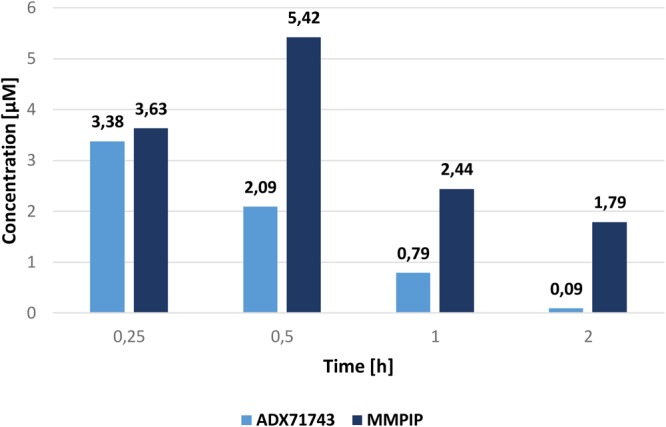
Comparison of ADX71743 and MMPIP concentrations in brain.

The concentration of ADX71743 and MMPIP in mouse plasma and brain are shown in **Table 1**. C_max_ was evident in brain and plasma 0.25 h after injection of ADX71743, and 0.5 h after MMPIP administration. **Figure 3** represents comparison between ADX71743 and MMPIP concentrations in the brain in selected time points after administration.

**Table 1:** Plasma **(A)** and brain **(B)** concentration of MMPIP and ADX71743 after administration of 10 mg/kg.

(A)		
**Parameters**	**ADX71743**	**MMPIP**
Tmax (h)	0.25	0.25
T1/2 (h)	0.90	1.16
Cmax (μmol/L)	3.73	9.85
AUC (μmol/L^∗^h)	1.90	13.52
**(B)**		
**Parameters**	**ADX71743**	**MMPIP**
Tmax (h)	0.25	0.50
T1/2 (h)	0.34	1.75
Cmax (μmol/L)	3.38	5.42
AUC (μmol/L^∗^h)	2.27	8.98

*Tmax – time at maximum observed concentration Cmax noted in minutes after administration of drug, T1/2 – terminal elimination half-life after administration, Cmax – maximum drug concentration obtained after administration of a drug between the time of doing and the final observed point, and AUC – the area under the concentration-time curve.*

Data presented in **Table 2** showed that ADX71743 and MMPIP had different cytochrome P450 inhibition profile. Weak inhibition (IC_50_ > 10μM) of cytochrome P450 was observed in case of 1A2, 2B6, 2C9, 2D6 isoforms for both NAM mGluR_7_ standards. Mild inhibition (3.3 < IC_50_ < 10) of isoform 2C19 was determined for ADX71743 standard, while strong inhibition (IC_50_ < 1.1) was observed only for MMPIP in case of isoform 3A4 as well as 2C19.

**Table 2:** *In vitro* profiles, physicochemistry, and ADME.

Parameters	ADX71743	MMPIP
Molecular weight	269.14	333.35
clogD	3.64	1.79
clogD	3.64	1.79
PSA	43.10	68.46
Kinetic solubility in HHB medium	509.87	5.6
Metabolic stability (microsomes, mice)	0.01	49.78
Clint	500.14	38.74
**Cytochrome P_450_ (IC_50_, μM)**		
1A2	> 10	> 10
3A4	> 10	< 1.1
2B6	> 10	> 10
2C9	> 10	> 10
2C19	3.3 < IC_50_ < 10	< 1.1
2D6	> 10	> 10

*Kinetic solubility (μg/ml), metabolic stability mice microsomes (% remaining after 60 min), and clint (ml/min/mg/ protein).*

### Electrophysiology

**FIGURE 4:**
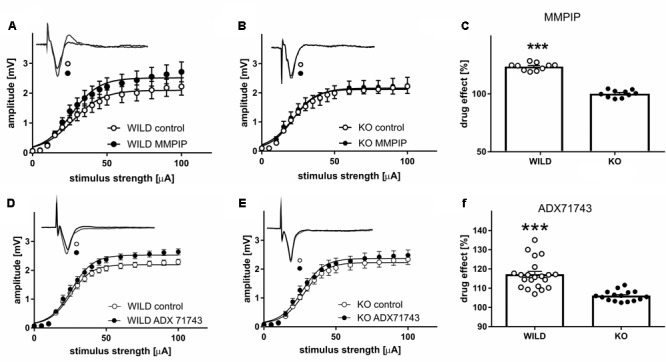
The influence of mGlu_7_-selective antagonist (MMPIP) on the relationship between stimulus intensity and amplitude of field potentials (FP) in WILD **(A)** and KO mice **(B)**, and the before-after effect of the drug **(C)**. The influence of mGlu_7_-selective antagonist (ADX71743) on the relationship between stimulus intensity and amplitude of FPs in WILD **(D)** and KO mice **(E)**, and the before-after effect of the drug **(F)**. Filled circles, quadrates: FPs recorded in slices after 20 min MMPIP/ADX administration prepared from WILD mice, open circles/quadrates: control preparations (*n* = 18). ^∗∗∗^*P* < 0.001.

Analyses of FPs recorded in slices obtained from WILD mice revealed an increase in the relationship between stimulus intensity and FP amplitude (input–output curve) after MMPIP administration in wild animals (**Figure 4A**), compared to KO (*P* < 0.001, paired *t*-test, **Figure 4B**). Parameters characterizing input-output curves of FPs, calculated using the Boltzmann fits, are summarized in **Table 3A**. The amplitude of FPs was markedly higher over a wide range of stimulation intensities (*P* < 0.001, two-tailed) (**Figure 4A**).

**Table 3:** Parameters characterizing input-output curves of FPs, calculated using the Boltzmann fits for MMPIP **(A)** and ADX71743 **(B)**.

(A)				
**Treatment**	**Vmax**	**Uh**	**S**	***n***
Veh	2.11 ± 0.2	27.37 ± 1.6	10.28 ± 0.8	10
Veh MMPIP	2.53 ± 0.3^∗∗∗^	26.93 ± 1.6	9.37 ± 0.83	10
KO	2.11 ± 0.18	21.64 ± 1.8	7.3 ± 0.2	4
KO MMPIP	2.16 ± 0.2	22.1 ± 1.4	8.6 ± 1.01	4
**(B)**				
**Treatment**	**Vmax**	**Uh**	**S**	***n***
Veh	2.195 ± 0.09	25.44 ± 1.2	6.42 ± 0.3	24
Veh ADX 71743	2.531 ± 0.1^∗∗∗^	26.07 ± 1.3	6.53 ± 0.3	24
KO	2.225 ± 0.17	27.19 ± 0.93	7.49 ± 0.4	16
KO ADX 71743	2.36 ± 0.18	26.06 ± 1.18	7.12 ± 0.41	16

*^∗∗∗^*P* < 0.001. Two-tailed paired *t*-test, *t* = 8.084, df = 9.*

*^∗∗∗^*P* < 0.001. Two-tailed paired *t*-test, *t* = 9.426, df = 23. Vmax is the maximum FP amplitude; Uh is the stimulation intensity evoking FP of half-maximum amplitude; S is the factor proportional to the slope of the curve; and *n* is the number of slices.*

The effect of MMPIP administration (before-after effect) was about 23% higher in WILD group compared to KO animals (123% vs. 100,1%, *P* < 0.001, two-tailed, *t* = 6.544 df = 30, **Figure 4C**).

ADX71743 administration increased the amplitude of recorded FP in wild animals, whereas were ineffective in KO group (*P* < 0.002; *P* < 0.49, paired *t*-test). Parameters characterizing input-output curves of FPs, calculated using the Boltzmann fits, are summarized in **Table 3B**. The amplitude of FPs increased over a higher ranges of stimulation intensities (*P* < 0.001, paired *t*-test, two-tailed, *t* = 9.426 df = 23) (**Figures 4D,E**). The effect of ADX71743 administration (before-after effect) was higher in WILD group compared to KO animals (116% vs. 106, *P* < 0.001, two-tailed, *t* = 5.71 df = 35, **Figure 4F**).

### MK-801-Induced Hyperactivity

**FIGURE 5:**
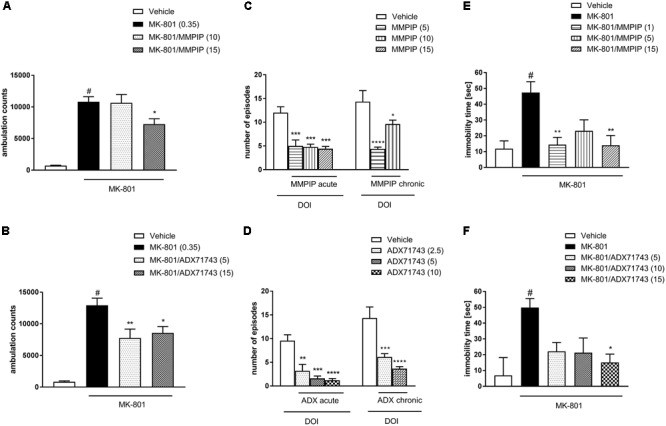
Effects of MMPIP and ADX71743 on MK-801-induced hyperactivity in mice that had been habituated to locomotor activity cages **(A,B)**, DOI-induced head twitches after acute (left panel) or chronic (right panel) administration **(C,D)**, and the immobility time in the modified forced swim test after chronic administration of MK-801 (13 days) **(E,F)**. Doses in mg/kg are indicated in parentheses. Data are presented as means ± SEM. ^#^*P* < 0.001 comparing to vehicle-treated animals, ^∗^*P* < 0.05, ^∗∗^*P* < 0.01, ^∗∗∗^*P* < 0.001, and ^∗∗∗∗^*P* < 0.0001 compared with the DOI or MK-801 treated group. Number of animals in each group *n* = 7 **(C,D)** or *n* = 10 **(A,B,E,F)**.

One-way ANOVA analysis revealed the statistically significant effects of treatments [*F*_(3.34)_ = 23.38, *P* < 0.0001 (**Figure 5A**) and *F*_(3.32)_ = 21.2, *P* < 0.0001 (**Figure 5B**)]. Neuman-Keuls *post hoc* analysis indicated significant increase in the locomotor activity after MK-801 administration when compared to control groups (*P* < 0.0001) and the significant reversal of MK-801-induced effect after MMPIP administration at the highest dose (15 mg/kg) used in the study (*P* < 0.05). Both doses of ADX71743 (5 and 15 mg/kg) decreased MK-801-induced hyperactivity in a statistically significant way (*P* < 0.001 and *P* < 0.05) when compared to MK-801-treated groups.

### DOI-Induced Head Twitches

One-way ANOVA analysis revealed the statistically significant effects of treatments [*F*_(3.16)_ = 13.96, *P* < 0.0001 (**Figure 5C**) and *F*_(3.18)_ = 15.75, *P* < 0.0001 (**Figure 5D**)]. Dunnet’s *post hoc* analysis indicated that the administration of MMPIP significantly reduced DOI-induced head twitches at all investigated doses 5, 10, and 15 mg/kg (*P* < 0.001) (**Figure 5C**). The effect of ADX71743 was also significant at 2.5 mg/kg (*P* < 0.01), 5 mg/kg (*P* < 0.001), and 10 mg/kg (*P* < 0.0001) (**Figure 5D**).

The compounds showed similar activity after chronic (10 days) administration: MMPIP at the doses 5 and 10 mg/kg and ADX71743 at the doses 2.5 and 5 mg/kg. One-way ANOVA analysis revealed the statistically significant effect of MMPIP treatment [*F*_(2.19)_ = 15.67, *P* < 0.0001] and Dunnet’s *post hoc* comparison revealed statistically significant effect of both doses (*P* < 0.05 and *P* < 0.0001). Similarly, the effect of ADX71743 administration was also significant [*F*_(2.17)_ = 16.56, *P* < 0.0001] and Dunnet’s *post hoc* comparison revealed the statistical effect of both investigated doses (*P* < 0.001 and *P* < 0.0001) (**Figures 5C,D**).

### Modified Forced Swim Test

One-way ANOVA analysis revealed the statistically significant effects of treatments [*F*_(4.44)_ = 5.8, *P* < 0.0007 (**Figure 5E**) and *F*_(4.45)_ = 4.3, *P* < 0.005 (**Figure 5F**)]. Neuman-Keuls *post hoc* analysis indicated significant increase in the immobility time after MK-801 administration when compared to control groups (*P* < 0.001) and the significant reversal of MK-801-induced effect after MMPIP administration at the dose of 1 mg/kg (*P* < 0.01) and 15 mg/kg (*P* < 0.01). The effect of ADX71743 was significant only at the dose of 15 mg/kg (*P* < 0.05) when compared to MK-801-treated group.

### Social Interaction Test

**FIGURE 6:**
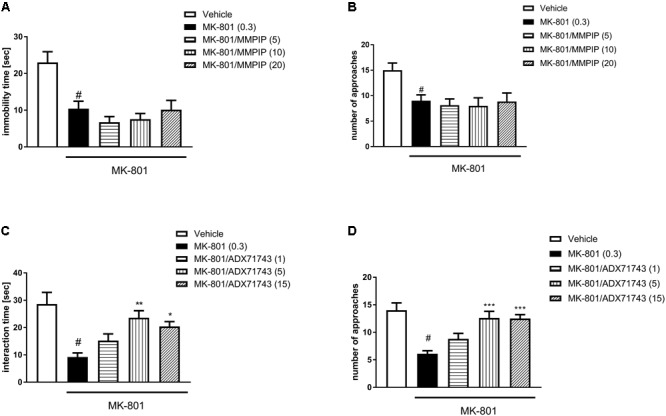
Effects of MMPIP **(A,B)** ADX71743 **(C,D)** on MK-801-induced social interaction deficits. Doses in mg/kg are indicated in parentheses. Data are presented as means ± SEM. ^#^*P* < 0.01 compared with the control group, ^∗^*P* < 0.05, ^∗∗^*P* < 0.01, and ^∗∗∗^*P* < 0.001 compared with the MK-801-treated group. Number of animals in group varied *n* = 8–10.

One-way ANOVA analysis revealed the statistically significant effects of treatment in the time of interaction [*F*_(4.30)_ = 9.20, *P* < 0.0001 and *F*_(4.45)_ = 7.63, *P* < 0.0001] (**Figures 6A,C**) and the number of episodes [*F*_(4.30)_ = 4.2, *P* < 0.007 and *F*_(4.45)_ = 10.44, *P* < 0.0001] (**Figures 6B,D**). Neuman-Keuls *post hoc* analysis indicated significant reduction of social behaviors after MK-801 administration when compared to control groups (*P* < 0.01). MMPIP had no effect on both measured parameters (**Figures 6A,B**).

ADX71743 at a dose of 5 and 15 mg/kg reversed the effect of MK-801 on the duration (*P* < 0.01 and *P* < 0.05) and number of social episodes (*P* < 0.001 and *P* < 0.001) (**Figures 6C,D**).

### Novel Object Recognition Test

**FIGURE 7:**
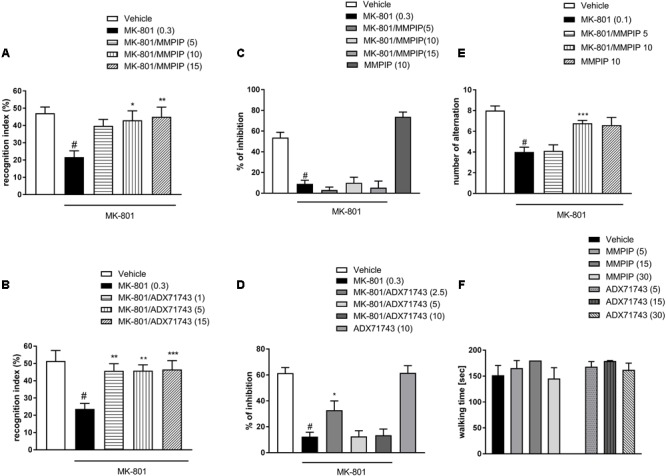
Effects of MMPIP and ADX71743 on MK-801-induced deficits in the NOR test **(A,B)** and prepulse inhibition **(C,D)**. The graph showing the effect of MMPIP on MK-801-induced disruption in spatial delayed alteration test **(E)** and the effect of MMPIP and ADX71743 on rotarod performance **(F)**. Doses in mg/kg are indicated in parentheses. Data are presented as means ± SEM. ^#^*P* < 0.001 compared with the control group, ^∗^*P* < 0.05, ^∗∗^*P* < 0.01, and ^∗∗∗^*P* < 0.001 compared with the MK-801-treated group. Number of animals in each group *n* = 8–10.

One-way ANOVA analysis revealed the statistically significant effects of treatments [*F*_(4.37)_ = 3.7, *P* < 0.01 (**Figure 7A**) and *F*_(4.44)_ = 5.99, *P* < 0.0006 (**Figure 7B**)]. Neuman-Keuls *post hoc* analysis indicated significant reduction of recognition index after MK-801 administration when compared to control groups (*P* < 0.01) and the significant reversal of MK-801-induced effect after MMPIP administration at the doses of 10 (*P* < 0.05) and 15 mg/kg (*P* < 0.01) (**Figure 7A**) and ADX71743 at the doses 1 mg/kg (*P* < 0.01), 5 mg/kg (*P* < 0.01), and 15 mg/kg (*P* < 0.001) (**Figure 6B**) when compared to MK-801-trated animals.

### Prepulse Inhibition

MK-801 (0.3 mg/kg) enhanced the amplitude of the acoustic startle response and markedly attenuated the prepulse-induced inhibition of the acoustic startle response (up to 16% of control). One-way ANOVA analysis revealed statistically significant effect of treatment [*F*_(4.39)_ = 22.84, *P* < 0.0001 (**Figure 7C**) and *F*_(4.39)_ = 19.96, *P* < 0.000 (**Figure 7D**)]. Neuman-Keuls *post hoc* comparison revealed that MK-801 inhibited startle response comparing to control groups (*P* < 0.0001). The effect of MK-801 on the prepulse-induced inhibition of the acoustic startle response was not antagonized by the selective NAM of mGlu_7_ receptor MMPIP in all doses (5, 10, and 15 mg/kg). When given alone, MMPIP (10 mg/kg) attenuated the amplitude of the acoustic startle response and markedly enhanced the prepulse-induced inhibition of the acoustic startle response (up to 137% of control), but the effect was not statistically significant (**Figure 7C**). ADX71743 inhibited MK-801-induced disruption in PPI at the lowest dose 2.5 mg/kg (*P* < 0.05) (**Figure 7D**).

### Spatial Delayed Alternation Test

One-way ANOVA analysis revealed a statistically significant effect of treatment [*F*_(4.42)_ = 10.59, *P* < 0.0001] (**Figure 7E**). Neuman-Keuls *post hoc* analysis indicated significant reduction of choice accuracy after MK-801 administration (*P* < 0.0001). MMPIP at a dose of 10 mg/kg rescued the MK-801–induced cognitive impairments, by improving the choice accuracy (*P* < 0.001) (**Figure 7E**).

### Motor Coordination

In the rotarod test, neither MMPIP nor ADX71743 did not induce detectable motor impairments when compared to the control group [*F*_(6.63)_ = 0.919] (**Figure 7F**).

## Discussion

The present paper constitutes the complex study concerning putative antipsychotic-like activity of mGlu_7_ receptor NAMs. We used two commercially available compounds, MMPIP and ADX71743. The first reports concerning their pharmacological activity were released several years ago, however, the activity of the compounds is still not fully investigated and established.

Here, we have used a variety of techniques that allowed us to investigate the *in vitro* and *in vivo* activity of both compounds, their pharmacokinetics and pharmacological effects.

Using T-REx 293 cell lines (commercially available version of HEK 293 cell line with single FRT site) with inducible expression of mGlu_7_ receptor the selectivity of the compounds toward mGlu_7_ receptors was confirmed. Both compounds dose-dependently antagonized L-Glu inhibition of cAMP accumulation in the presence of forskolin and L-glutamate. Similar results were shown earlier for ADX71743 (Kalinichev et al., 2013).

This is the first report showing the activity of MMPIP on HEK line, and thus can be compared with the activity of ADX71743. The earlier studies with MMPIP were performed on CHO lines, and the activity of the compound was investigated in the presence of L-AP4, without the presence of L-glutamate. Additionally, different techniques for intracellular cAMP assessment like AplhaScreen and Phenyx cAMP assays were used to characterize both NAMs which can differ in sensitivity and measurement range (Suzuki et al., 2007; Kalinichev et al., 2013). In the Kalinichev et al.’s (2013) paper, HEK 293 cells and L-glutamate were used. The EC_50_ for L-Glu and IC_50_ for ADX71743 in the presence of EC_80_ the agonist were significantly lower comparing to results obtained by our group. Different method of cAMP measurements and different host cells caused difficulties to collate the biological activity of the two chemicals. Here, we compare the activity of both compounds and our result indicates that the affinity of MMPIP is slightly better than that of ADX71743, although both compounds are very potent. Moreover, we demonstrated inverse agonist action for both MMPIP and ADX71743 in heterologous expression system. Our results and data presented by Suzuki et al. (2007) confirmed the intrinsic activity of mGluR_7_ that can be showed in the presence of inverse agonist. Moreover, this may have very important biological effect on *in vivo* studies due to dual way of action of ADX71743 and MMPIP. However, we must keep in mind differences which can be observed between species and even between different cell hosts from the same species. For example, the mGluR_7_ positive allosteric modulator AMN082 activates the receptor in CHO cells, as well its effect can be observed in behavioral studies. In contrast, human cell line HEK 293 expressing mGluR_7_ does not respond to this compound (Niswender et al., 2010).

Systemic administration of the compounds confirmed that they reach Cmax rapidly, 0.25–0.5 h after administration, followed by a rapid decline. The concentration of ADX71743 was almost undetectable 2 h after administration, while the concentration of MMPIP was still observed. Similar pharmacokinetic profile was described earlier for the compounds; however, the doses of ADX71743 used were much higher (100–150 mg/kg) than that used in our studies, and the compound was administered s.c. (Kalinichev et al., 2013), while MMPIP was administered similarly as in the work of Hikichi et al., 2010. The important thing is that in the present studies several time points were analyzed in contrary to the work of Hikichi et al. (2010) where the concentration of the compound was measured only at one time point, 1 h after administration (Hikichi et al., 2010). Comparing the results obtained for both compounds it may be concluded that the most potent NAM of mGluR_7_ – ADX71743 exhibits high kinetic solubility, low metabolic stability in mice liver microsomes consistent with high clearance, while MMPIP shows better metabolic stability but lower biological activity as well as solubility.

Subsequently, the specificity of compounds was assessed in electrophysiology experiments, in which, with the use of mGlu_7_ KO mice, we established that the compounds were active only on the slices obtained from wild type animals and not from mGlu_7_ KO mice. Then, we compared the activity of the compounds in variety of behavioral models. In our earlier studies, the propsychotic effect of mGlu_7_ PAM, AMN082, was showed (Wierońska et al., 2012). Therefore, it could be assumed that NAMs of mGlu_7_ receptor can be proposed as putative antipsychotic agents. To confirm this hypothesis, both compounds were examined in variety of animal tests and models with high predictive validity toward antipsychotic-like efficacy of drugs.

MK-801 induced hyperactivity and DOI-induced head twitches were used as the tests predictive for positive symptoms of schizophrenia. Both compounds reversed MK-801-induced deficit without exerting own effects on spontaneous locomotor activity in active doses. The activity of ADX71743 was more evident in this test, and lower doses of the compound (5 mg/kg) restored MK-801-induced deficit. MMPIP was active only in the highest administered dose, 15 mg/kg. In previous studies, the activity of ADX71743 was showed in amphetamine-induced hyperactivity, but much higher doses were needed to reach significant effect (100 and 150 mg/kg were active). Also, the different route of administration (subcutaneous) was applied in that studies (Kalinichev et al., 2013). The activity of MMPIP has not been investigated in this paradigm so far.

Both investigated compounds also reversed DOI-induced head twitches. DOI, similar to the other 5-HT_2A_ activating agents (i.e., d-lysergic diethylamide acid, LSD), has hallucinogenic potential in humans (Jacobs and Trulson, 1979; Geyer and Vollenweider, 2008; Vollenweider and Kometer, 2010), and in animals, it induces characteristic head twitches (González-Maeso et al., 2008; De Gregorio et al., 2016a), that are reversed by the administration of both typical and atypical neuroleptics (Marona-Lewicka et al., 2005; De Gregorio et al., 2016b). The activity of ADX71743 was observed in lower doses than that observed after MMPIP administration. Again our results differ from the results presented in the studies of Kalinichev et al. (2013) where ADX71743 was active at the dose of 100 mg/kg and higher.

In the next step, the activity of both drugs was investigated in tests for negative symptoms of schizophrenia, such as social interaction and modified forced swim test. The social interaction test resembles social withdrawal observed in schizophrenia patients while modified forced swim test is considered as a model of depressive-like symptoms of schizophrenia (Noda et al., 1995, 1997; de Moura Linck et al., 2008). In both tests, only atypical (e.g., risperidone), and not typical neuroleptics, effectively reverse MK-801-induced deficits (de Moura Linck et al., 2008). It is in line with clinical efficacy of drugs, where only atypical neuroleptics are potent to reverse negative symptoms, although the efficacy of drugs is not always satisfactory.

Here, both compounds were active in modified forced swim test and only ADX71743 reversed MK-801-induced deficits in social interaction test. So again the activity of ADX71743 was better than the activity of MMPIP.

Novel object recognition (NOR) and spatial delayed alteration (SDA) were used as the models of cognition, while PPI reflects attentional deficit associated with schizophrenia. MK-801 disrupts the ability of animals to discriminate between the known, old object, and the novel one in NOR (Nilsson et al., 2007; Grayson et al., 2015), and to make proper choice to obtain the reward in SDA. This disruption of short working memory is antagonized by atypical, but not typical antipsychotics. In NOR, both drugs were active, but the activity of ADX71743 was more evident. Additionally, MMPIP was tested in SDA test and prevented, in all investigated doses, the disruptive effect of MK-801. In the former studies performed in the work of Hikichi et al. (2010), the drug was shown to reduce the recognition index in the NOR test and decreased the location index in the object location test at the doses 10–30 mg/kg. Therefore, it seems that the compound does not possess any procognitive effect when given alone, on contrary it rather disturbs cognitive behaviors, while when given prior MK-801 it prevents the development of disruptive effects of the drug. The other compound, ADX71743, was not investigated in the models of cognition yet. Therefore, this is the first study showing the pro-cognitive activity of the compound in the models of schizophrenia. Here, we also show for the first time that ADX71743 in low doses is potent to prevent MK-801-induced disruption in PPI, while such an activity was not observed for MMPIP, similarly as in previous studies (Hikichi et al., 2010).

In these studies, the activity of compounds was well-investigated in pharmacologically induced animal models of schizophrenia, showing their preventive effect on MK-801-induced disruptions of those behaviors after acute administration. However, it should be taken into consideration that the potential desensitization effect, after repeated administration of two compounds, may be responsible for the failure in neuroleptic efficacy in clinical trials. Therefore both compounds were administered chronically to compare if their efficacy will be similar as after acute administration. The activity of the compounds was tested in DOI-induced head twitches and no tolerance was observed. However, further studies are needed especially with non-pharmacologically induced animal model of schizophrenia to fully characterize the antipsychotic properties of above described compounds.

**FIGURE 8:**
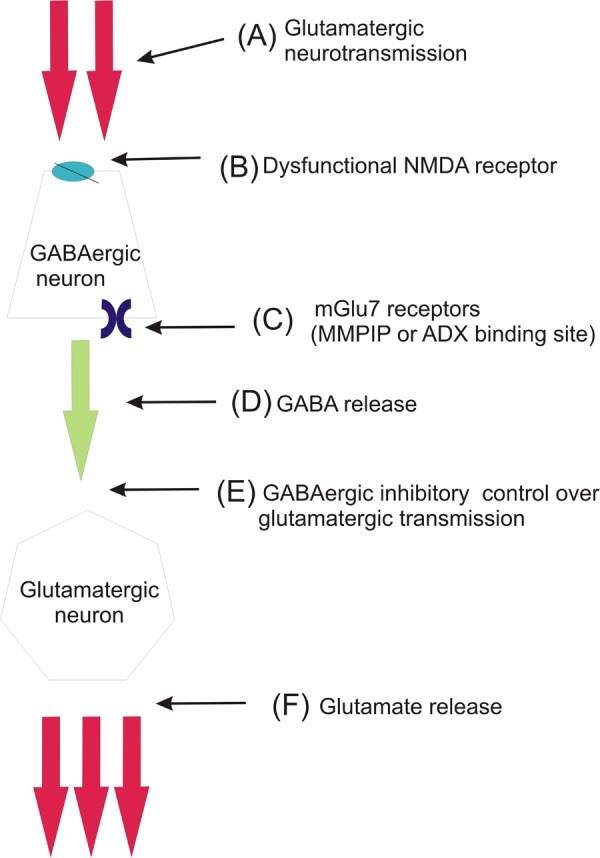
Schematic mechanism involving the role of mGlu_7_ receptor in schizophrenia pathogenesis (hypothesis partially based on Conn et al., 2009). In normally functioning brain, glutamate **(A)** stimulates GABAergic interneurons via NMDA receptors **(B)** to release GABA **(D)**, which in turn exerts inhibitory control over thalamocortical glutamatergic innervation **(E)**. In schizophrenia this inhibitory control of glutamatergic neurotransmission is lost due to dysfunction of NMDA receptors **(B)** expressed on GABAergic cell bodies. This leads to enhanced glutamate release from thalamocortical glutamatergic neurons **(F)**. The inhibition of mGlu_7_ receptors, which are expressed presynaptically on GABAergic neurons by MMPIP or ADX **(C)**, leads to activation of GABA release and restores the GABAergic inhibitory control over glutamatergic neurons.

Considering the putative mechanism of action of mGlu_7_ NAMs, it must be taken into consideration that mGlu_7_ receptors are localized mainly on GABAergic terminals (Dalezios et al., 2002). The expression of this receptor on GABAergic neurons is almost 10 times higher than on glutamatergic neurons. Therefore, the receptor predominantly regulates GABA release than glutamate release (Summa et al., 2013). Its activation leads to inhibition of GABA release while its inhibition may contribute to increased release of this neurotransmitter. According to the hypothesis of schizophrenia raised by Conn et al. (2009), the increased release of glutamate due to the loss of inhibitory control over the glutamatergic neurons is the main cause of schizophrenia development. The majority of recent studies concerning antipsychotic activity of mGlu ligands was focused on the inhibition of glutamate release through the activation of the receptors expressed on glutamatergic nerve terminals. Here, it seems that the inhibition of mGlu_7_ receptors expressed on GABAergic neurons may contribute to the increase of GABA efflux and thus bring back the inhibitory control over the glutamatergic transmission (scheme of the **Figure 8**).

Based on the present studies, it is clear that the investigated compounds may have preventive effect in developing psychotic behaviors. However, to better establish the role of mGlu_7_ receptor in schizophrenia and putative antipsychotic effects of its inhibition, more work must be undertaken and new ligands with better pharmacokinetic properties acting at mGlu_7_ receptor should be synthesized. The trend is now open as recently a paper was released, where new compounds inhibiting mGlu_7_ receptor were proposed (Reed et al., 2017).

## Ethics Statement

All the procedures were conducted in accordance with the European Communities Council Directive of September 22, 2010 (2010/63/EU) and Polish legislation acts concerning animal experimentations.

## Author Contributions

PC and MW performed the DOI-induced head twitches, social interaction, and NOR. PC contributed to the analysis of the results and manuscript writing. KK carried out the pharmacokinetics studies. PB and GB performed the cAMP analysis. AC performed the PPI. BB performed the electrophysiology experiments. PG and EL performed the SDA. AP-P performed the hyperactivity and modified forced swim test. AW performed the analysis of video recordings from behavioral tests (SI, NOR). AP collected the funds and contributed to the discussion. JW performed the data analysis, coordination, wrote the manuscript, and collected the funds.

## Conflict of Interest Statement

The authors declare that the research was conducted in the absence of any commercial or financial relationships that could be construed as a potential conflict of interest.
